# Serologic Cross-Reactivity with Pandemic (H1N1) 2009 Virus in Pigs, Europe

**DOI:** 10.3201/eid1601.091190

**Published:** 2010-01

**Authors:** Constantinos S. Kyriakis, Christopher W. Olsen, Susy Carman, Ian H. Brown, Sharon M. Brookes, Jan Van Doorsselaere, Kristien Van Reeth

**Affiliations:** Ghent University, Ghent, Belgium (C.S. Kyriakis, K. Van Reeth); University of Wisconsin, Madison, Wisconsin, USA (C.W. Olsen); University of Guelph, Guelph, Ontario, Canada (S. Carman); Veterinary Laboratories Agency-Weybridge, Addlestone, UK (I.H. Brown, S.M. Brookes); KATHO Catholic University of South-West Flanders, Roeselare, Belgium (J. Van Doorsselaere)

**Keywords:** Viruses, influenza, pigs, Europe, North America, pandemic (H1N1) 2009, serology, cross-reaction, expedited, dispatch

## Abstract

We tested serum samples from pigs infected or vaccinated with European swine influenza viruses (SIVs) in hemagglutination-inhibition assays against pandemic (H1N1) 2009 virus and related North American SIVs. We found more serologic cross-reaction than expected. Data suggest pigs in Europe may have partial immunity to pandemic (H1N1) 2009 virus.

Pandemic (H1N1) 2009 virus is a reassortant of >2 circulating swine influenza viruses (SIVs) (*1*). Six gene segments, including the gene encoding a classical H1 hemagglutinin (HA), are similar to ones found in triple reassortant SIVs circulating in pigs in North America. The neuraminidase (NA) and matrix genes originate from Eurasian avian-like H1N1 SIV. This specific reassortant had never been found in swine when it was first detected in humans. However, during May–October 2009, pandemic (H1N1) 2009 virus was isolated from swine in Canada, Argentina, Australia, Singapore, (Northern) Ireland, Norway, the United States, Japan, and Iceland (*2*). Humans were suspected to be the source of infection; pigs did not contribute to spread of the virus in humans.

Recent experimental studies confirmed pandemic (H1N1) 2009 virus can become established in swine populations (*3*,*4*). A key question is whether immunity to circulating SIVs, which differ antigenically and genetically in different parts of the world, protects pigs against pandemic (H1N1) 2009 virus. Three SIV subtypes are cocirculating in European swine populations: an avian-like subtype H1N1 virus introduced from wild birds in 1979, a human-like subtype H3N2 reassortant with HA and NA genes acquired from descendants of Hong Kong/68 pandemic virus, and a subtype H1N2 reassortant virus, which acquired H1 from human influenza (H1N1) viruses in the 1980s (*5*). We aimed to clarify whether pigs infected or vaccinated with these viruses have cross-reactive antibodies against pandemic (H1N1) 2009 virus or related North American SIVs, both of which have an antigenically distinct, classical H1 HA.

## The Study

We tested 66 swine sera obtained from previous experimental studies (*6*). All pigs were negative for SIV antibodies at the start of the experiments. Serum samples were collected 3–4 weeks after the last infection or immunization. Immunization categories, experimental groups, and influenza virus strains used are shown in the Table.

Twenty-seven pigs were intranasally (IN) inoculated with 7.0 log_10_ 50% egg infectious doses of swine/Belgium/1/98 (avian-like swine influenza [H1N1]), swine/Gent/7625/99 (reassortant influenza [H1N2] with human-lineage HA), swine/Flanders/1/98 (reassortant influenza [H3N2] with human-lineage HA), or some combination of the three. These viruses are representative of prevailing SIVs in western Europe. Two groups received a single inoculation with either SIV (H1N1) or SIV (H1N2). Three groups received dual, consecutive inoculations with 2 SIV subtypes at a 4-week interval.

Twenty-four pigs received 2 intramuscular (IM) doses of commercial, inactivated SIV vaccine, at a 4-week interval. One vaccine contained a classical swine-lineage subtype H1N1 virus, A/New Jersey/8/76; the remaining 3 contained various avian-like subtype H1N1 SIVs (Table). The vaccines contained various types of adjuvants.

**Table:** Serologic cross-reactivity between North American H1 SIVs and pandemic (H1N1) 2009 virus after experimental infection and/or immunization of pigs by various methods with European SIVs*

Group	No. pigs	Range of antibody titers of positive animals (no. pigs with HI antibody titers ≥10)							
		European SIVs				North American H1 SIVs			Pandemic (H1N1) 2009 cH1†
		Swine/ Belgium/ 98 (H1N1) avH1†	Swine/ Gent/99 (H1N2) huH1†	Swine/ Flanders/98 (H3N2) huH3†		Swine/ Iowa/2004 (H1N1) cH1†	Swine/ Ontario/ 2004 (H1N1) cH1†	Swine/ Indiana/99 (H1N2) cH1†	
Infection‡									
H1N1 (avH1†)	6	40–160 (6)	–§	–		–	–	–	10 (1)
H1N2 (huH1†)	6	–	40–160 (6)	–		–	–	–	–
H1N1 (avH1†)- 4w - H1N2 (huH1†)	5	80–160 (5)	20–40 (5)	–		20 (4)	10–20 (5)	10–20 (5)	20–40 (5)
H1N2 (huH1†)- 4w - H1N1 (avH1†)	4	80–160 (4)	160–320 (4)	10 (3)		10–40 (4)	20–80 (4)	20–40 (4)	20–160 (4)
H1N1 (avH1†)- 4w - H3N2 (huH3†)	6	40–160 (6)	–	20–80 (6)		–	10–40 (3)	10–20 (4)	10–40 (6)
Double vaccination¶									
New Jersey/8/76 (H1N1, cH1†)	6	10–40 (6)	10 (1)	10–80 (5)		80–160 (6)	20–80 (6)	80–320 (6)	10–20 (5)
Swine/Netherlands/25/ 80 (H1N1, avH1†)	6	10–40 (6)	10 (1)	10–160 (6)		20–40 (5)	10–80 (6)	10–40 (6)	10 (3)
Swine/Belgium/230/92 (H1N1, avH1†)	6	20–640 (6)	10–20 (2)	20–320 (6)		10–320 (5)	10–320 (6)	10–160 (5)	10–640 (5)
Swine/Haselunne/2617/ 2003 (H1N1, avH1†)	6	10–40 (6)	40–160 (6)	20 (6)		–	20 (1)	10–20 (5)	10 (1)
Infection# followed by single vaccination									
H1N1 (avH1†)- 4w - New Jersey/8/76	6	640–2,560 (6)	10 (3)	10 (1)		40–80 (6)	40–80 (6)	80–320 (6)	40–80 (6)
Hyperimmunization**									
H1N1 (avH1†)	3	320–1,280 (3)	10–20 (2)	–		40–160 (3)	40–160 (3)	40–80 (3)	40–160 (3)
H1N2 (huH1†)	3	40 (1)	320–1,280 (3)	–		20 (1)	10–80 (2)	40 (1)	80 (1)
H3N2 (huH3†)	3	–	–	160–640 (3)		–	–	–	–

*SIV, swine influenza virus. †Phylogenetic lineage of the hemagglutinin gene: c, classical swine; hu, human; av, avian. ‡Infection with swine/Belgium/1/98 (H1N1), swine/Gent/7625/99 (H1N2), or swine/Flanders/1/98 (H3N2). §Antibody titers <10 in all pigs. **¶**Only the influenza (H1N1) vaccine component is shown. The first 3 vaccines are bivalent (subtypes H1N1, H3N2); the fourth is trivalent (subtypes H1N1, H3N2, H1N2). #Infection with swine/Belgium/1/98 (H1N1). **Each individual pig was hyperimmunized with a different European SIV isolate: subtype H1N1 viruses were swine/Finistère/2899/82, swine/Belgium/1/98, and swine/Gent/132/2005; subtype H1N2 viruses were swine/Scotland/410440/94, swine/Gent/7625/99, and swine/Gent/177/2002; subtype H3N2 viruses were swine/Gent/1/84, swine/Flanders/1/98, and swine/Gent/131/2005.

Six pigs were first inoculated IN with swine/Belgium/1/98 (H1N1) followed by a single IM administration of New Jersey/8/76-based SIV vaccine 5 weeks later. Nine pigs were hyperimmunized against various European SIVs (Table) by IN inoculation, followed by an IM innoculation with the same virus in combination with Freund’s complete adjuvant 4 weeks later.

All serum samples were examined in hemagglutination-inhibition (HI) assays against the European subtype H1N1, H1N2, and H3N2 viruses listed above; 3 North American SIVs with a classical H1; and A/California/04/2009, a prototype pandemic (H1N1) 2009 virus (*7*). The North American SIVs included swine/Iowa/H04YS2/2004 (triple reassortant influenza [H1N1]), swine/Ontario/11112/04 (reassortant influenza [H1N1]), and swine/Indiana/9K035/99 (triple reassortant influenza [H1N2]). Low amino acid homology was present in the HA1 region of the HA gene between the European H1 SIVs used for infection of pigs and the North American H1 SIVs (range 70%–75%) or the pandemic (H1N1) 2009 virus (69%–72%). When compared with avian-like influenza (H1N1) strains in European vaccines, the New Jersey/8/76 vaccine strain was more closely related to North American SIVs (77%–81% vs. 92%–94%) and pandemic (H1N1) 2009 virus (72%–75% vs. 90%).

HI antibodies induced by a single infection with European subtype H1N1 or H1N2 SIVs did not cross-react with North American H1 SIVs or with pandemic (H1N1) 2009 virus. In contrast, consecutive infections with 2 European subtypes frequently induced cross-reactive antibodies, even though European viruses do not contain a classical swine H1 HA.

Three European vaccines also induced cross-reactive antibodies to North American H1 SIVs, and 2 induced antibody titers >20 to pandemic (H1N1) 2009 virus in most pigs. HI titers against pandemic virus were lower with A/New Jersey/8/76-based vaccine than with vaccine containing an avian-like SIV (H1N1).

A single vaccination with A/New Jersey/8/76-based vaccine induced only minimal HI antibody titers in influenza-naive pigs, but existing antibody titers (data not shown) to European avian-like subtype H1N1 SIV in pigs previously infected with this virus dramatically increased, even though these viruses contain HA proteins of the avian and classical swine lineages respectively. All pigs had antibodies to the viruses with a classical swine H1 HA, including pandemic (H1N1) 2009 virus.

Finally, hyperimmunization with European SIVs of subtype H1N1, H3N2, or H1N2 resulted in high titers to the homologous viruses. Cross-reactions with viruses with a classical H1 were consistently observed with serum samples from pigs hyperimmune to avian-like European influenza (H1N1) viruses, but they were rare or absent after hyperimmunization with subtype H1N2 or H3N2 strains.

## Conclusions

A large antigenic and genetic distance exists between European H1 SIVs and viruses with a classical H1 HA (*8*,*9*). Nevertheless, pigs infected or vaccinated with European SIVs frequently have cross-reactive HI antibodies to pandemic (H1N1) 2009 virus and related North American SIVs. Two factors predispose for serologic cross-reactivity: 1) elevated antibody titers to European avian-like subtype H1N1 SIVs, as in hyperimmune swine serum and some postvaccination serum; and 2) infection with 2 European SIV subtypes.

Consecutive experimental infection of pigs with the antigenically distinct SIVs of subtypes H1N1 and H1N2 causes a strong boost of already existing HI antibody titers to the first infecting virus, as shown by longitudinal investigations (*6*). These pigs may even develop low levels of cross-subtype-reactive HI antibodies to SIV (H3N2). In humans and in mice, sequential infections with influenza virus variants seemingly lead to a predominant antibody response against cross-reactive epitopes on the HA, which are shared with the first infecting virus, whereas the response to strain-specific epitopes may be lower (*10*). Such cross-reactive antibodies may cause cross-recognition of viruses with a classical H1 HA in our dually infection–immune pigs, but further studies with monoclonal antibody escape mutants are needed to understand cross-reactivity at the molecular level. Multiple SIV infections have become common in swine-dense regions of Europe since the introduction of the H1N2 subtype in the late 1990s (*11*).

The HI test will clearly fail to differentiate here between established SIVs and pandemic (H1N1) 2009 virus. This conclusion is further supported by preliminary HI tests on sera collected from finishing pigs in Belgium during 2007. Of 172 serum samples, 35% and 36%, respectively, were positive against swine/Iowa/2004 (H1N1) and swine/Indiana/99 (H1N2); 95% of these positives had antibodies to >1 European SIV subtype.

Besides serum antibody to the HA, an infection with live influenza virus also stimulates mucosal immunity and cellular immune responses to highly conserved viral epitopes (*12*). These responses mean partial protection against a subsequent infection with an antigenically unrelated strain may occur in the absence of cross-reactive antibodies (*13*). In contrast, protection offered by inactivated influenza vaccines is almost entirely dependent on serum HI antibody titers. Two of the 4 vaccines used induced HI antibody titers against pandemic (H1N1) 2009 virus that are considered protective. Unexpectedly, avian influenza (H1N1) SIV-based vaccine gave higher antibody titers than the A/New Jersey/76-based vaccine. The amino acid homology between vaccine and test strains is thus a less reliable predictor of serologic cross-reactivity than one would assume. Our data suggest that preexisting immunity to established SIV strains may partially protect pigs in Europe against pandemic (H1N1) 2009 virus, but the extent of such protection needs to be assessed in well-controlled challenge experiments. Pandemic (H1N1) 2009 infection in pigs in Europe has so far been limited to countries where SIVs are absent or circulating at low levels. Whether the virus will become established in countries where SIVs are widespread remains to be seen.

The divergence between H1 of contemporary seasonal influenza (H1N1) viruses of humans and pandemic (H1N1) 2009 virus is approximately the same as that between SIVs in Europe and North America. However, cross-reactive HI antibody responses to pandemic (H1N1) 2009 virus have been almost exclusively detected in humans born before 1950 (*14*). These persons have been exposed to older variants of seasonal influenza (H1N1) viruses that are more closely related to classic swine influenza (H1N1) viruses and pandemic (H1N1) 2009 virus. The present epidemiologic situation in humans thus differs from that in pigs. Two antigenically distinct influenza (H1N1) viruses—seasonal and the 2009 virus—could cocirculate in humans in the future. This cocirculation will likely broaden serologic responses and protection against influenza but may also complicate interpretation of HI test results.
